# Structural Analysis of Specific Metal Chelating Inhibitor Binding to the Endonuclease Domain of Influenza pH1N1 (2009) Polymerase

**DOI:** 10.1371/journal.ppat.1002831

**Published:** 2012-08-02

**Authors:** Eva Kowalinski, Chloe Zubieta, Andrea Wolkerstorfer, Oliver H. J. Szolar, Rob W. H. Ruigrok, Stephen Cusack

**Affiliations:** 1 European Molecular Biology Laboratory, Grenoble Outstation, BP181, Grenoble, France; 2 Unit of Virus Host-Cell Interactions, UJF-EMBL-CNRS, UMI 3265, BP181, Grenoble, France; 3 Savira pharmaceuticals Gmbh, Vienna, Austria; Johns Hopkins University - Bloomberg School of Public Health, United States of America

## Abstract

It is generally recognised that novel antiviral drugs, less prone to resistance, would be a desirable alternative to current drug options in order to be able to treat potentially serious influenza infections. The viral polymerase, which performs transcription and replication of the RNA genome, is an attractive target for antiviral drugs since potent polymerase inhibitors could directly stop viral replication at an early stage. Recent structural studies on functional domains of the heterotrimeric polymerase, which comprises subunits PA, PB1 and PB2, open the way to a structure based approach to optimise inhibitors of viral replication. In particular, the unique cap-snatching mechanism of viral transcription can be inhibited by targeting either the PB2 cap-binding or PA endonuclease domains. Here we describe high resolution X-ray co-crystal structures of the 2009 pandemic H1N1 (pH1N1) PA endonuclease domain with a series of specific inhibitors, including four diketo compounds and a green tea catechin, all of which chelate the two critical manganese ions in the active site of the enzyme. Comparison of the binding mode of the different compounds and that of a mononucleotide phosphate highlights, firstly, how different substituent groups on the basic metal binding scaffold can be orientated to bind in distinct sub-pockets within the active site cavity, and secondly, the plasticity of certain structural elements of the active site cavity, which result in induced fit binding. These results will be important in optimising the design of more potent inhibitors targeting the cap-snatching endonuclease activity of influenza virus polymerase.

## Author Summary

The 2009 influenza pandemic, the on-going potential threat of highly pathogenic H5N1 avian strains and the widespread occurrence of resistance to current anti-influenza drugs targeting the neuraminidase or the M2 ion channel, all highlight the need for alternative therapeutic options to treat serious influenza infections in the absence of protection by vaccination. The viral polymerase, which performs transcription and replication of the RNA genome, is an attractive target for novel antiviral drugs since potent polymerase inhibitors will directly stall replication. The heterotrimeric polymerase performs transcription by a unique cap-snatching mechanism, which involves host pre-mRNA cap-binding and endonucleolytic cleavage by the PB2 and PA subunits respectively. Crystal structures of both the PB2 cap-binding and PA nuclease domains are now available allowing structure-guided optimisation of cap-snatching inhibitors. Here we present a series of co-crystal structures of the 2009 pandemic H1N1 PA endonuclease domain that reveal the binding mode of several known endonuclease inhibitors. All inhibitors chelate the two manganese ions in the active site of the nuclease but different extensions to the metal binding scaffold bind in distinct sub-pockets of the active site cavity. These results highlight the value of structure-based approaches to the development of more potent influenza polymerase inhibitors.

## Introduction

Influenza virus replicates in the nucleus of infected cells where the heterotrimeric viral RNA-dependent RNA polymerase, with subunits PA, PB1 and PB2, is responsible for replication and transcription of the single-stranded viral RNA genome (vRNA). Transcription of viral mRNAs occurs through an unusual ‘cap-snatching’ mechanism [1] which has only been reported for negative strand, segmented RNA viruses, including orthomyxoviruses (notably influenza), bunyaviruses and arenaviruses. For influenza, cap-snatching involves the binding of host cell pre-mRNAs via their 5′ cap structure to the PB2 subunit of the polymerase followed by cleavage at nucleotides 10–13 by an endonuclease activity which resides in the PA subunit of the polymerase. The short capped oligomers then serve as primers for transcription of the viral mRNAs by the PB1 subunit of the polymerase. The viral transcripts are poly-adenylated by a stuttering mechanism at a conserved U-rich region of the template vRNA [2]; thus the viral mRNAs have both the 5′ and 3′ signals to be competent for translation after nucleo-cytoplasmic export. In the last few years, crystal structures of the two functional domains involved in cap-snatching have been determined (reviewed in [3]). The cap-binding domain resides in the central region of the PB2 subunit and has a unique fold while still binding the m^7^G ligand by means of an aromatic sandwich, similar to other cap-binding proteins [4]. The endonuclease domain is at the N-terminus of the PA subunit and has a core fold similar to other two-metal dependent nucleases of the PD…D/E…K superfamily [5]–[6]. Indeed, the isolated, recombinant endonuclease domain has divalent cation dependent, *in vitro* nuclease activity with a strong preference for manganese ions, consistent with the much tighter binding of manganese than magnesium [7]. Since transcription by cap-snatching is essential for virus replication, inhibition of either the cap-binding, endonuclease or polymerase activities are all potential means of anti-viral therapy and indeed each of these targets have been or are being actively pursued [8]–[10]. Indeed combination therapy targeting more than one of the polymerase active sites is an attractive possibility. Here, we exploit the availability of the endonuclease crystal structure to provide the first detailed structural information on specific inhibitor binding to the influenza polymerase.

The need for new therapeutic options targeting influenza virus is now widely recognised. This follows recent developments, such as the on-going circulation of highly pathogenic avian H5N1 strains, which could potentially adapt for human-to-human transmission [11], the unexpected emergence of the 2009 H1N1 pandemic strain [12], which was highly contagious and thus spread rapidly around the world, but was fortunately not so virulent, and the development of resistance in wild-type strains to currently available anti-viral drugs targeting the neuraminidase or M2 ion channel [13]. These have all highlighted the vulnerability of the world population to novel influenza strains for which there may be no vaccine for several months and a limited variety of resistance prone anti-viral drugs [14]. The cap-snatching endonuclease of influenza virus polymerase has been targeted for anti-influenza drug development since the 1990s because its inhibition would directly stall viral transcription and hence replication. Firstly, a number of 4-substituted 2,4-dioxobutanoic acid compounds that specifically inhibit influenza polymerase endonuclease activity with IC50s in the range 0.2 to 29 µM were identified by Merck [15]–[17]. Subsequently, a substituted 2,6-diketopiperazine natural compound (Flutimide) from the fungus *Delitschia confertaspora*, and its derivatives, were shown to inhibit endonuclease activity and influenza A and B virus replication in cell culture [18]. Bristol-Myers Squibb identified N-hydroxamic acid and N-hydroxyimide compounds that inhibit the endonuclease [19]. Roche discovered a new class of endonuclease inhibitors, with IC50s down to 3 µM based on considerations of the likely divalent cation binding properties of the enzyme [20]. More recently other compounds have been shown to inhibit the endonuclease, for instance green tea catechins [21], phenethylphenylphthalimide analogues derived from thalidomide [22] and macrocyclic bisbibenzyls [23].

The X-ray structure of the A/Victoria/3/1975(H3N2) endonuclease [5] was obtained with a crystal form in which crystal contacts blocked access to the active site. Despite many trials, no crystal structures have been obtained of H3N2 endonuclease with bound inhibitors, substrate or product analogues, severely limiting the possibility for structure-based inhibitor optimisation. To overcome this problem, we investigated whether the endonuclease from other influenza strains having slightly different amino acid sequences might yield more useful crystal forms. We found that a construct comprising residues 1–198 of A/California/04/2009(H1N1) (2009 pandemic strain, pH1N1), expressed from a synthetic gene, readily crystallised with and without relevant ligands. In the residue range 1–198 of the construct, the pH1N1 sequence differs in 12 positions from that of H3N2; however the active site and close vicinity are identical between the two strains. Here we report crystal structures of the pH1N1 influenza endonuclease complexed with two divalent metal ions and four 4-substituted 2,4-dioxobutanoic acid inhibitors [15]. The compounds are 2,4-dioxo-4-phenylbutanoic acid (DPBA), 4-[3-[(4-chlorophenyl)methyl]-1-(phenylmethylsulpho)-3-piperidinyl]-2-hydroxy-4-oxo-2-butenoic acid (denoted R05-01), 4-[1-cyclohexylmethyl-4-(p-chlorobenzyl)piperidin-4-yl]2,4-dioxobutanoic acid (denoted R05-02) and 4-[N-benzyl-3-(4-chlorobenzyl)piperidin-3-yl]2,4-dioxobutanoic acid (Merck L-735,882; here denoted R05-03) (Table 1). These compounds are reported to have IC50s of 21.3, 0.19, 0.33 and 1.1 µM respectively in an *in vitro* cap-dependent transcriptase assay [15]. A structure is also presented with bound (-)-epigallocatechin gallate (denoted EGCG, Table 1) from green tea. Two additional structures are presented of the pH1N1 endonuclease with bound mononucleotides rUMP and dTMP, components of the RNA (and DNA) endonuclease substrate. In an accompanying paper, structures of a complementary set of inhibitors bound to the endonuclease of strain A/Vietnam/1203/2004 (H5N1) are reported [24].

**Table 1:** Summary of inhibitor complexes crystallised, chemical description of ligands and crystallisation conditions.

Ligand	Protein	CA index name of ligand	Composition of ligand	Crystallisation reservoir condition	Space group (No. molecules per asymmetric unit)	Resolution/PDB code
Native	PA pH1N1	-	-	1.6 M sodium formate	*C*2 (4)	2.1 Å
	1-198			0.1 M HEPES pH7		4AVQ
				5% glycerol		
DBPA	PA pH1N1	2,4-dioxo-4-phenylbutanoic acid	C10 H8 O4	1.0 M LiCl_2_	*C*2 (4)	2.3 Å
	1–198			0.1 M HEPES pH 7		4AWF
				20% PEG6000		
R05-1	PA pH1N1	4-[3-[(4-chlorophenyl)methyl]-1-(phenylmethylsulpho)-3- piperidinyl]-2-hydroxy-4-oxo-2- butenoic acid	C23 H24 Cl N S O6	25–30% PEG 4K	*P* 6_2_22 (1)	1.9 Å
	1–198			0.1 M Tris pH8.5		4AWK
	del52–64:Gly			0.2 M NaCl		
R05-2	PA pH1N1	4-[4-[(4-chlorophenyl)methyl]-1-(cyclohexylmethyl)-4-piperidinyl]- 2-hydroxy-4-oxo-2-butenoic acid	C23 H30 Cl N O4	25% PEG 3350	*P*2_1_2_1_2_1_ (4)	2.2 Å
	1–198			0.1 M NH_4_SO_4_		4AVG
				0.1 M Bis-Tris pH5.5		
R05-3	PA pH1N1	4-[3-[(4-chlorophenyl)methyl]-1-(phenylmethyl)-3-piperidinyl]-2- hydroxy-4-oxo-2-butenoic acid	C23 H24 Cl N O4	2.0 M NH_4_SO_4_	*P*2_1_2_1_2_1_ (4)	2.6 Å
	1–198			0.1 M Bis-Tris pH5.5		4AWG
EGCG	PA pH1N1	[(2R,3R)-5,7-dihydroxy-2-(3,4,5- trihydroxyphenyl)chroman-3- yl]3,4,5-trihydroxybenzoate	C22 H18 O11	10% PEG 3350	*P*6_4_22 (1)	2.6 Å
	1–198			0.1 M NaCl		4AWM
	del52–64:Gly			0.1 M HEPES pH 7		
rUMP	PA pH1N1	5′-uridylic acid	C9 H13 N2 O9 P	25% PEG 3350	*P*2_1_2_1_2_1_ (4)	2.05 Å
	1–198			0.1 M NH_4_SO_4_		4AWH
				0.1 M Bis-Tris pH5.5		
dTMP	PA pH1N1	5′-thymidylic acid	C10 H15 N2 O8 P	25% PEG 3350	*P*2_1_2_1_2_1_ (4)	1.87 Å
	1–198			0.2 M NH_4_SO_4_		4AVL
				0.1 M Bis-Tris pH5.5		

Diagrams of each ligand are given in Figure 2.

All these structures show in detail how these compounds bind directly to the metal ions as well as interacting with a number of residues in the active site, some of which change conformation upon ligand binding. This three-dimensional knowledge of the ligand interacting residues and the regions of plasticity of the active site is critical for the optimised design of modifications to existing inhibitors to improve their potency or for structure based design and optimisation of novel inhibitors that effectively block endonuclease activity.

## Results

### Comparison of PA-Nter from pH1N1, H5N1 and H3N2 structures

**Figure 1:**
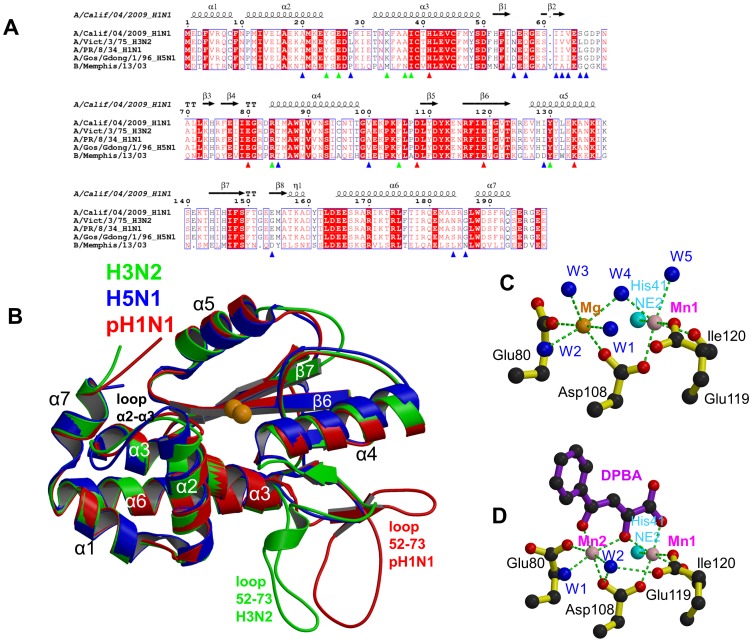
The PA endonuclease carries a divalent cation binding site in its active center. **A:** Sequence alignment of the PA-Nter endonuclease from four influenza A (including the three of known atomic structure) and one influenza B strain. The secondary structure of the pH1N1 domain is shown over the alignment. Red triangles indicate conserved cation binding (His41, Glu80, Asp108, Glu119) and catalytic (Lys134) residues. Blue triangles indicate naturally variable positions amongst influenza A strains. Green triangles indicate residues interacting with the inhibitors described in this paper. **B:** Superposition of PA endonuclease structure from H3N2 (green, PDB entry 2W69), H5N1 (blue, PDB entry 3EBJ) and pH1N1 (red, this work). The two bound divalent metal ions are represented by orange spheres. Flexible region 53–73 is at the bottom right and only ordered in certain chains from the H3N2 (B chain) and pH1N1 (e.g. D chain of dTMP complex) structures. For H5N1, region 53–73 is not visible. Major secondary structure elements are shown consistent with those in Figure 1A. **C:** Divalent ion co-ordination in the native endonuclease structure. Manganese and magnesium ions are respectively pink and orange spheres and co-ordinating water molecule blue spheres and the ion co-ordination is shown with green dotted lines. For clarity, only His41 NE2 is shown (cyan sphere). **D:** Divalent ion co-ordination in the DPBA bound structure. Manganese ions are pink spheres and co-ordinating water molecule blue spheres and the ion co-ordination is shown with green dotted lines. For clarity, only His41 NE2 is shown (cyan sphere).

Structures are now known of the PA-Nter from H3N2 [5], H5N1 [6] and pH1N1 (this work). There are respectively 5 and 12 differences between the pH1N1 sequence compared to the avian H5N1 and human H3N2 strain sequences, at a total of 15 positions (Figure 1A). It is not known whether any of these differences play a role in inter-species transmission or virulence. The most structurally variable region, which is also a hotspot for sequence variation (Figure 1A), is between residues 55–66. This forms part of a mobile inserted element (residues 53–73) of unknown function that is solvent exposed and usually disordered. In the H3N2 and pH1N1 structures, this element is well defined in some chains in the asymmetric unit and shows a significantly different orientation between the two strains (Figure 1B). However this could be due to different crystal contacts since in the H3N2 structure, loop residue Glu59 interacts with a divalent cation in the active site of a neighbouring molecule, thus blocking access to the active site in this crystal form (Figure S4 in [5]). To avoid the problems associated with this flexible loop, two of the six structures described below (those of R05-1 and EGCG) were determined using a truncated form of the protein in which residues 52–64 were replaced by a single glycine (Δ52–64:Gly). In an accompanying paper it is shown that an even larger loop deletion (Δ51–72:Gly-Gly-Ser) does not significantly affect enzymatic activity [24]. Apart from this, the three PA-Nter structures from different strains are overall very similar, with only small differences in helix orientation and loop conformation (Figure 1B).

### Divalent cation binding in the native structure

Previous structural studies on H3N2 endonuclease showed that there are two divalent cation binding sites in PA-Nter: Site 1 is coordinated by His41, Asp108, Glu119 and the carbonyl oxygen of Ile120 and site 2 by Glu80 and Asp108 [5]. However as mentioned above, the active site structure of H3N2 PA-Nter could have been influenced by crystal contacts. Subsequent *in vitro* studies showed that manganese ions bind strongly and exclusively to site 1 and that both manganese and magnesium ions can bind at site 2 albeit with significantly lower affinity [7]. A similar situation occurs in the related La Crosse bunyavirus endonuclease [25]. As the exact *in vivo* situation is unknown, all *in vitro* work reported here, notably crystallization and *in vitro* activity assays, was performed in buffer containing both 2 mM MnCl_2_ and 2 mM MgCl_2_. In the unliganded pH1N1 structure at 2.1 Å resolution (Tables 1), there is excellent definition of the solvated bi-metal binding site in each of the four crystallographically independent active sites. Both metal ions have octahedral co-ordination: site 1 by His41, Asp108, Glu119, Ile120 and two water molecules (W4 and W5) and site 2 by Glu80 and Asp108 and four water molecules (W1, W2, W3 and W4) (Figure 1C). Whereas site 1 refines as a fully occupied manganese ion, the metal in site 2 has weaker electron density and thus could correspond to a magnesium ion or partially occupied manganese. Indeed, refinement with a fully occupied magnesium ion in site 2 gives B-factors similar to that of the manganese ion in site 1. Since the anomalous signal in the original dataset was poor, we attempted to clarify this assignment by measuring additional crystallographic data at a longer X-ray wavelength of 1.55 Å, where the putative manganese anomalous signal is higher. In this 2.6 Å resolution dataset, a strong anomalous signal is observed in site 1 in each of the four active sites (between 7.4 and 10.1σ, data not shown), whereas the electron density is much weaker or even absent for site 2 and has no significant anomalous scattering. These results provide structural confirmation of our previous biochemical studies that indicated strong binding of manganese to site 1 and weaker binding of either magnesium or manganese to site 2 [7].

### Characterisation of compound binding and anti-viral activity

All diketo compounds and ECGC were tested for their *in vitro* nuclease inhibitor and *in cellulo* antiviral activities by a fluorescence resonance energy transfer (FRET) assay and cell viability assay, respectively (Table 2). An alternative *in vitro* fluorescence polarization assay for nuclease inhibition has recently been described elsewhere [26]. For the FRET assay, a single stranded RNA oligonucleotide labeled with an emitter and quencher fluorophore at opposite ends was incubated with A/Victoria/3/1975(H3N2) PA-Nter with and without inhibitors. RNA cleavage was monitored by the increase in fluorescence when the quencher is released from the emitter (Figure S1A). IC50 values of 2.7 µM for DBPA, 1.9 µM for EGCG, 1.1 µM for R05-03, 0.13 µM for R05-01, and 0.06 µM for R05-02 were obtained with this method (Figure S1B). In the cell viability assay, in which MDCK cells were infected with influenza virus and treated with endonuclease inhibitor at the same time, most compounds could inhibit virus replication thereby preventing virus induced cytopathicity and restoring cell viability compared to a virus infected control sample. DBPA did not show any inhibitory effect whereas IC50 values of 19.9 µM for R05-03, 20.4 µM for R05-01, 15.9 µM for R05-02 and 1.1 µM for EGCG were obtained (Table 2). EGCG and R05-3 showed some meaurable cytotoxicity (Table 2). The 15–20-fold lower IC50 for EGCG in this assay compared to the diketo compounds R05-01 and R05-02, whereas in the nuclease inhibition assay, the reverse was true, might be due to the different physicochemical properties, and hence cell availability, of the substances tested. It should be noted that the systematically lower IC50 values quoted in the introduction for the diketo compounds referred to an *in vitro* transcriptase assay, not an anti-viral assay [15]. In addition to the functional assays, the effect of the diketo inhibitors on the thermal stability of the endonuclease was tested by a Thermofluor assay in which a hydrophobic fluorophore has little affinity for native proteins but binds to denatured proteins, leading to an increase of fluorescence [27]. The apparent melting temperature (T_m_) of denaturation can be obtained from the temperature dependence of the fluorescence (Figure S1C). It has previously been shown that PA-Nter is significantly thermally stabilized by divalent cation binding and, even more so, by DPBA binding [5], [7]. Here we report Tm values of 53.5, 65, 69, 71 and 69°C for no ligand, DPBA, R05-01, R05-02 and R05-03 respectively (Table 2). This confirms that R05-01, R05-02 and R05-03 all chelate the cations in the active site of the endonuclease and enhance the thermal stability even more than for DPDA, presumably by making additional stabilizing interactions with the protein.

**Table 2:** Endonuclease inhibition (FRET), anti-viral effect (CPE), cytotoxic dose and thermo-stabilisation (Tm) of pH1N1 PA-Nter by diketo compounds and EGCG.

Compound	FRET^a^ IC50 [µM]	antiviral activity IC50 [µM]^b^	CC50^c^ [µM]	Tm^e^ [°C]
none	-	-	-	53.5
DPBA	2.7±0.3	inactive	>200^d^	65
R05-1	0.13±0.04	20.4±3.0	>100^d^	69
R05-2	0.06±0.02	15.9±4.0	>50^d^	71
R05-3	1.1±0.5	19.9±2.0	79.8±11.5	69
EGCG	1.9±0.4	1.1±0.4	54.7±4.5	nd^f^

a Fluorescence resonance energy transfer (FRET) based endonuclease activity assay. FRET measurements were determined in quadruplicates with at least two independent sets. Mean values and 95% confidence limits (as indicated) were calculated.

b Reduction of virus induced cytopathic effect (CPE); inactive: no antiviral activity at the highest concentration tested. For CPE IC50 and CC50 values samples were applied in duplicates and IC50 values, CC50 values, and corresponding 95% confidence intervals were determined. The experiments were independently performed at least twice.

c 48 h cytotoxicity on MDCK cells.

d No cytotoxicity at the highest concentration tested (mainly limited by solubility).

e Apparent melting temperature Tm derived from Thermofluor measurements.

f Not determined due to fluorescence quenching.

### DPBA bound structure

The DPBA bound structure was determined at 2.3 Å resolution in the same *C*2 space-group as the native protein (Tables 1 and 3) and is shown in Figure 1D (electron density in Figure S2A). As with the native structure, anomalous scattering confirms the presence of manganese in site 1 (Figure S2A), whereas the metal in site 2 has a higher B-factor and no significant anomalous scattering. As expected, DPBA binds directly to the two cations bound in the active site. Compared to the unbound state, metal ion coordinating water molecules W3, W4 and W5 are replaced by oxygens from the ligand (Figures 1C,D). An identical configuration was observed for DPBA binding to the active site of bunyavirus cap-snatching endonuclease [25]. The catalytic Lys134 makes an electrostatic interaction with the carboxyl-group of DPBA, which also interacts with the hydroxyl of Tyr130 via a bridging water molecule. Ligand stabilisation of the metal binding to the active site almost certainly explains why DPBA supershifts the endonuclease thermal stability [5]. The phenyl ring of the DPBA is less well-defined than the rest of the molecule as its rotational conformation is only weakly stabilized by partial stacking with the side-chain of Arg84. An identical conformation of DPBA bound to A/Vietnam/1203/2004 (H5N1) PA-Nter is reported in the accompanying paper although this structure contains a second DPBA molecule in the active site stacking against the first, probably as a result of the high concentration used [24].

**Table 3:** Crystallographic data collection and refinement statistics.

Ligand	Native	DPBA	R05-1	R05-2	R05-3	EGCG	dTMP	rUMP
Protein	pH1N1 PA 1–198	pH1N1 PA 1–198	pH1N1 PA 1–198 Δ52–64:Gly	pH1N1 PA 1–198	pH1N1 PA 1–198	pH1N1 PA 1–198 Δ52–64:Gly	pH1N1 PA 1–198	pH1N1 PA 1–198
Wavelength (Å)	0.9395	0.9474	0.9724	0.9724	0.8726	0.8726	0.9765	0.9724
Space Group	*C*2	*C*2	*P*6_2_22	*P*2_1_2_1_2_1_	*P*2_1_2_1_2_1_	*P*6_4_22	*P*2_1_2_1_2_1_	*P*2_1_2_1_2_1_
Cell dimensions (Å)	a = 263.63 b = 66.24 c = 66.32 β = 95.98°	a = 260.87 b = 65.75 c = 66.03 β = 95.76°	a = b = 75.06 c = 120.05 γ = 120°	a = 56.59 b = 120.81 c = 128.20	a = 54.57 b = 122.54 c = 129.78	a = b = 99.9 c = 82.7 γ = 120°	a = 54.89 b = 121.24 c = 129.02	a = 54.96 b = 120.20 c = 128.07
Resolution range* (Å)	50-2.1 (2.2-2.1)	50-2.3 (2.4-2.3)	50-1.9 (1.95-1.90)	50-2.2 (2.3-2.2)	50-2.6 (2.7-2.6)	50-2.6 (2.7-2.6)	50-1.87 (1.92-1.87)	50-2.05 (2.10-2.05)
Completeness* (%)	99.0 (97.9)	97.3 (95.2)	99.9 (100)	99.6 (99.8)	99.8 (99.5)	98.3 (96.7)	99.8 (99.9)	99.5 (99.7)
R-sym* (%)	9.3 (65.8)	9.5 (39.7)	4.7 (53.7)	6.8 (51.8)	15.5 (81.2)	15.3 (83.9)	6.0 (86.9)	4.8 (50.7)
I/sigmaI*	10.63 (2.2)	7.45 (2.16)	22.19 (2.95)	14.68 (2.44)	9.50 (2.14)	12.07 (3.14)	16.83 (2.22)	11.36 (1.62)
No. of unique reflections used in refinement (free)	63353 (2643)	46575 (1899)	15580 (830)	42983 (2306)	26117 (1374)	7434 (363)	68174 (3655)	51176 (2750)
R-factor*	0.235 (0.320)	0.224 (0.296)	0.208 (0.297)	0.196 (0.258)	0.190 (0.262)	0.223 (0.354)	0.198 (0.307)	0.207 (0.270)
R-free *	0.284 (0.362)	0.289 (0.431)	0.236 (0.337)	0.252 (0.385)	0.262 (0.388)	0.278 (0.574)	0.235 (0.327)	0.243 (0.331)
**No.of atoms**: Total	6575	6548	1575	6785	6525	1579	6647	6672
Protein	6397	6235	1449	6446	6232	1481	6321	6367
Ligand	-	4×DPDA (56)	1×R05-1 (32	4×R05-2 (116)	4×R05-3 (116)	2×EGCG (66)	4×dTMP (84)	4×UMP (84)
Metal ions	4×(Mg^2+^, Mn^2+^)	4×(Mn^2+^, Mn^2+^)	1×(Mn^2+^, Mn^2+^)	4×(Mn^2+^, Mn^2+^)	4×(Mn^2+^, Mn^2+^)	1×(Mn^2+^, Mn^2+^)	4×(Mn^2+^, Mn^2+^)	4×(Mn^2+^, Mn^2+^)
Solvent	170	249	92	181	99+14×SO_4_	30	234	213
Mean B-value (Å^2^) (all atoms)	35.4	28.1	39.0	36.9	35.2	44.2	48.5	40.2
**Ramachandran plot (Molprobity)**								
Favoured (%)	96.5	98.0	98.2	97.4	95.5	96.5	97.0	96.3
Allowed (%)	99.9	100	100	99.9	99.7	100	99.7	99.7
Disallowed (%)	0.1	0	0	0.1	0.3	0	0.3	0.3
**RMS deviations from ideal values**								
Bond distances(Å)	0.014	0.013	0.008	0.017	0.013	0.008	0.013	0.010
Angles (°)	1.55	1.54	1.17	1.836	1.57	1.18	1.48	1.29

*: Last shell in brackets.

### Diketo inhibitor bound structures

As summarised in Table 3, co-crystals with compounds R05-2 and R05-3 were obtained with native protein in the *P*2_1_2_1_2_1_ space-group with four molecules in the asymmetric unit. For R05-01, crystallisation was successful with the Δ52–64:Gly truncation mutant in the *P*6_2_22 space-group. All diketo inhibitors co-ordinate the two metal ions in the same manner as described for DPBA and refinement is consistent with the presence of two bound manganese ions as confirmed by anomalous scattering (Figures S2B, S2D and S2E). In general, for each of the diketo compounds, the ‘arms’ have higher B-factors than the metal binding, diketo moiety of the ligand, suggesting flexibility due to sub-optimal interactions of the arms. Indeed, bound R05-03 is observed in two different conformations, corresponding to different rotamers of the chlorobenzene, in respectively chains A, B (denoted conformation 3A, Figure S2B) and C, D (denoted conformation 3D, Figure S2C) in the asymmetric unit. Conformation 3A has stronger electron density than 3D. For R05-02 electron density for the arms is not well-defined in all four copies in the asymmetric unit, although the configuration of the compound is unambiguous and consistent in each copy (Figure S2D). As discussed in more detail below, the two ‘arms’ of each diketo compound sample different sub-pockets of the active site cavity.

**Figure 2:**
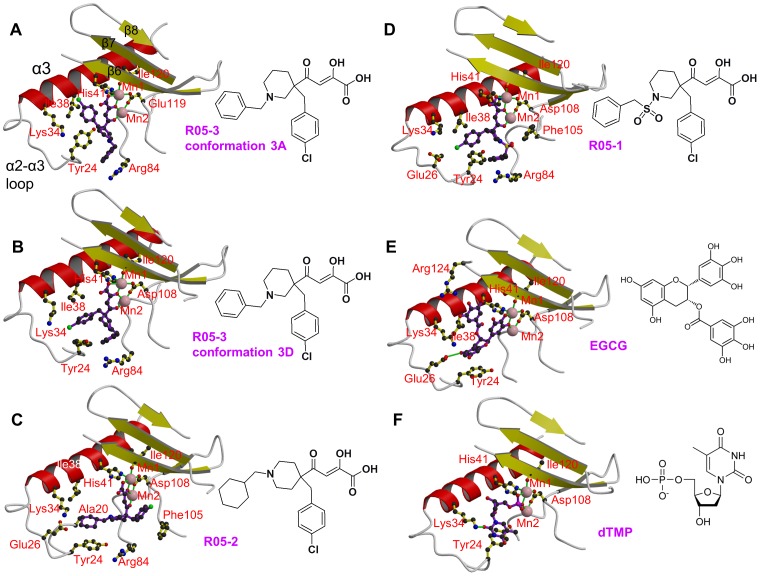
Binding of diketo inhibitors, EGCG and dTMP in the active site of pH1N1 endonuclease. Manganese ions are pink spheres and the ion co-ordination is shown with green lines. Side chains of key active site residues that interact with the compound or are close to it are shown. The orientation in each case is the same after superposition of the domain. Helix α3 (red), the α3-α3 loop and beta strands β6, β7 and β8 (yellow) are indicated in panel A. **A:** R05-3 in conformation 3A. **B:** R05-3 in conformation 3D. **C:** R05-2 (chain A). Ala20 is marked in addition (see discussion). **D:** R05-1. **E:** EGCG. **F:** dTMP.

Figures 2A–D show a comparison of the binding mode of R05-01, R05-02, R05-03A and R05-03D in the active site indicating nearby residues. Most interactions of the compound arms are with residues in the range 24–38 (notably Tyr24, Glu26, Lys34, Ala37 and Ile38), which comprise a flexible loop leading from the C-terminal end of helix α2 into the N-terminal half of helix α3, but Arg84 and Phe105 are also involved in some cases. Due to the hydrophobic and aromatic nature of the arms, most interactions are van der Waals or stacking and there are no polar interactions.

### EGCG-bound structure

Epigallocatechin 3-gallate (EGCG) is the ester of epigallocatechin and gallic acid and is the most abundant catechin in green tea. EGCG, a polyphenol with antioxidant properties, has been extensively investigated as a possible antiviral or anticancer compound [28]–[29]. It has recently been reported that EGCG inhibits the influenza endonuclease [21]. Co-crystallisation of the pH1N1 PA-Nter Δ52–64:Gly truncation mutant with EGCG gave a new crystal form diffracting to 2.6 Å resolution (Table 1,3). The compound was clearly observed in the active site as well as anomalous scattering peaks corresponding to the two manganese ions (Figure S3A). Strong extra density also exists around a 2-fold crystallographic axis and represents another EGCG molecule non-specifically trapped by crystal packing. The conformation and placement of the EGCG in the active site is shown in Figure 2E with more details of the interactions shown in Figure S3B. The two manganese ions are co-ordinated by two of the hydroxyls of the gallo-group, whilst the galloyl-group is orientated towards helix α3, stacking on Ala37 and Ile38 and hydrogen bonding to the carbonyl oxygen of Val122. The planes of the gallo- and galloyl-phenyl groups are parallel but not significantly overlapped. The double ring of EGCG is orientated towards the preceding loop, with notably the resorcinol moiety stacking on Tyr24 and making a hydrogen bond to Glu26. However three of the eight hydroxyl groups of EGCG do not make direct interactions with the protein. The configuration of the EGCG in the active site is quite different from that previously proposed by docking studies [21], [23].

### rUMP/dTMP bound structures

**Figure 3:**
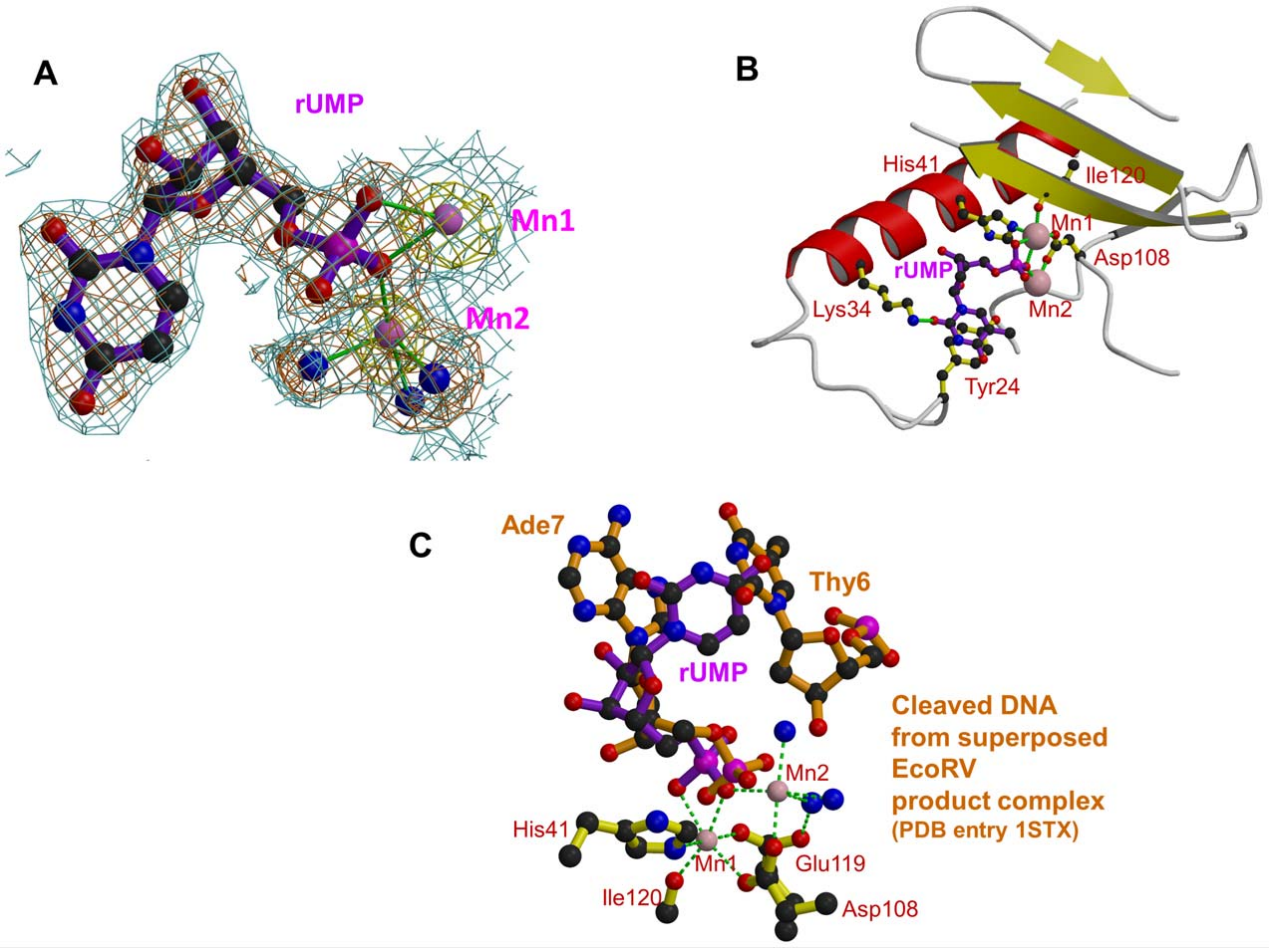
Binding of dTMP/rUMP in the active site of pH1N1 endonuclease. **A:** Electron density for rUMP in pH1N1 PA endonuclease. Manganese ions are pink spheres, co-ordinating water molecule blue spheres and the ion co-ordination is shown with green lines. Blue contour: final 2Fo-Fc electron density at 1.0σ. Brown contour: Fo-Fc unbiased difference map at 2.8σ. Yellow contour: anomalous density at 4.0σ. **B:** Binding site of rUMP in the active site following the same scheme as in Figure 2. **C:** rUMP bound in the active site of pH1N1 PA (purple) with superposed DNA from product complex of EcoRV (brown, pdb entry 1STX). Active site residues (yellow), manganese ions (pink) and water molecules (blue) are for the rUMP structure. The position of the two DNA bases either side of the cleavage site in the EcoRV product complex is shown.

Co-crystallisation trials were attempted with all four deoxy- and oxy- mononucleotides, to mimic putative substrate binding by the endonuclease. The only compounds that resulted in structures were dTMP and rUMP, both of which gave large, well-ordered crystals in a new orthorhombic space-group (Tables 1 and 3). Apart from the obvious differences in the ribose and base, the two structures are essentially the same. In both cases, clear anomalous scattering exists for the two manganese ions (Figure 3A) and the nucleotides bind with two oxygens of the phosphate completing the co-ordination sphere of Mn1, one of them also coordinating Mn2 (Figure 2F, 3A, 3B). The base is well stacked on Tyr24 and Lys34 makes a hydrogen bond to the O2 position. The ribose is stacked on Ala37 and Ile38 of helix α3 and the hydroxyl groups do not make hydrogen bonds to the protein. This is consistent with the fact that the protein is a DNAase as much as an RNAase [5], [30]. The conformation we observe for rUMP is quite different from that previously published (PDB entry 3hw3 [31]). The latter structure was obtained by soaking nucleotides into existing crystals of the endonuclease in the absence of manganese and the electron density is very poor. In this structure, a water molecule replaces Mn1 and a magnesium ion replaces Mn2. This difference in metal ligation is reflected in the altered conformation of Glu119. The ribose and base positions are quite different from in our structure and unable to interact with Lys34 or Tyr24 (for comparison of the structures see Figure S4). We suspect that the differences between the two structures reflect firstly the lack of manganese and secondly the fact that soaking pre-grown crystals does not allow the active site to adapt to the ligand as is more likely the case for co-crystallisation.

Unlike some of the diketo inhibitors, dTMP/rUMP exhibits a very well defined, full occupancy binding mode. The apparent optimisation of this binding might reflect its biological significance as representing part of the natural nucleic acid substrate binding site. It has previously been shown that superposing the active sites of PA-Nter with EcoRV restriction enzyme closely overlaps the metal binding centre and catalytic lysine (see [5]). To examine this further, we superposed various complexes of EcoRV with bound substrate or product dsDNA complexes. As seen in Figure 3C, the bound rUMP most closely mimics the position of the post-cleavage nucleotide as observed in EcoRV PDB structure 1STX [32]. A preference for uridine in the natural substrate at the post-cleavage position has not been reported before, although it has been proposed that in infected cells cleavage of donor pre-mRNA preferentially occurs after Cyt-Ade [33] or, alternatively, Gua-Cyt [34]. Further biochemical and structural work is clearly required on PA-Nter substrate or product complexes to advance understanding of any intrinsic sequences preferences of PA-Nter and the exact mechanism of cleavage.

## Discussion

### Sub-pockets and plasticity of the active site cavity

**Figure 4:**
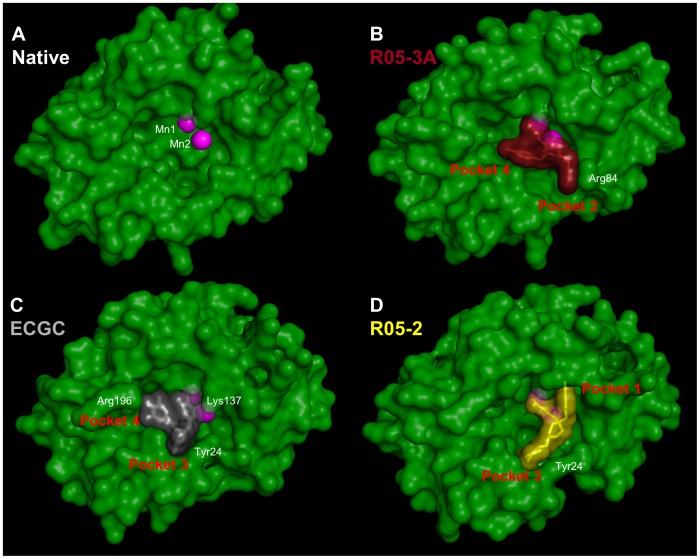
Active site pockets of the PA endonuclease. Each panel shows the pH1N1 domain in surface representation (green) in the same orientation with the manganese ions as pink spheres. Bound compounds are shown in surface and stick representation. Some residues underlying prominent surface features are indicated in white. **A**. Native unliganded structure. **B**. R05-3A (brick). **C**. EGCG (grey). **D**. R05-2 (yellow). In the native state, the active site cavity is large. The different compounds fill various sub-pockets of the cavity (indicated in red) and induced fit movements tend to close up the active site, but the cavity is never entirely filled.

**Figure 5:**
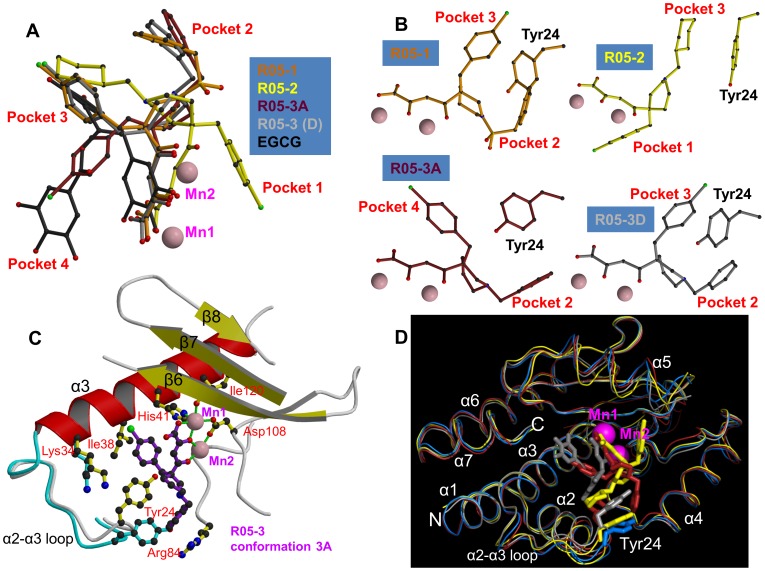
Active site plasticity of the PA endonuclease. **A:** Superposition of diketo inhibitors and EGCG bound in the PA active site after structural alignment of the entire endonuclease domain for each structure. R05-1 in orange, R05-2 in yellow, R05-3 conformation 3A in brick, R05-3 conformation 3D in light grey and EGCG in black. Manganese ions are pink spheres. The aromatic extensions from the metal binding scaffold are inserted into different sub-pockets of the active site. **B:** Configuration of the diketo inhibitors (coloured as in Figure 3A) with respect to residue Tyr24. All panels are in the same orientation. **C:** Diagram comparing the native (cyan) and R05-3 bound form in the conformation 3A (purple). Tyr24 side-chain moves to partially stack on the chlorobenzene and Arg84 is re-ordered to stack with the benzene ring of R05-3A. Manganese ions are pink spheres and the ion co-ordination is shown with green lines. **D:** Superposition of the Cα-trace of native (cyan) and R05-3A (brick), R05-2 (yellow) and EGCG (grey) pH1N1 structures. Manganese ions are pink spheres. Much of the structure, notably the metal binding catalytic center, is relatively rigid, but there is more flexibility in the α2-a3 loop (especially at Tyr24) and also in the α5 helix (which bears the catalytic lysine).

The active site cavity of the endonuclease is quite voluminous, presumably because it has to accommodate at least two nucleotides either side of the cleavage site, with the manganese ions at its back (Figure 4A). As shown in the superposition of Figure 5A, the metal chelating moiety of the three diketo and EGCG inhibitors binds in a similar orientation to the manganese ions (although compound R05-2 is slightly tilted) but the two ‘arms’ of each compound are inserted into combinations of different active site ‘pockets’, denoted pockets 1 to 4 (see also Figure 4B–D). R05-1 has a similar configuration to R05-3D, with the two arms occupying pockets 2 and 3. R05-3A occupies pockets 2 and 4. Compound R05-2, which differs from R05-1 and R05-3 in the point of substitution on the piperidinyl ring (Figure 2) occupies pocket 3 and, uniquely, pocket 1. The green tea compound EGCG and the mononucleotides occupy pockets 3 and 4. Pocket 1 allows stacking on Phe105 and is uniquely observed with the chlorobenzene of R05-2. Pocket 2 is characterised by stacking on the side-chain of Arg84 (e.g. benzene of R05-3A, R05-3D and R05-1). Pocket 3 is characterised by stacking on Tyr24 (chlorobenzene of R05-3D, R05-1, cyclohexane of R05-2, resorcinol moiety of ECGC, base of rUMP/dTMP). Occupation of pocket 4 is characterised by stacking on Ile34 (e.g. chlorobenzene of R05-3A, galloyl-group of EGCG, rUMP/dTMP ribose). These various stacking options may reflect the need for similar interactions with the bases of the RNA substrate, as also suggested by the observed stacking of rUMP on Tyr24.

Figure 5B illustrates the conformational changes and ordering that occur upon R05-03 binding, in particularly of the loop around Tyr24, which is poorly ordered in the native structure. Indeed, Tyr24 is observed to be in three rotamers depending on which of the four pockets are occupied (Figure 5C). Side-chains of other residues (e.g. Arg84, Lys34, Glu26, Phe105) also change depending on which pockets the ligand is occupying. This indicates a plasticity of the active site and an induced fit mode of ligand binding, which is a complication that needs to be taken into account in any *in silico* screening for putative inhibitors. A second important conclusion for designing more potent inhibitors is to ensure that the extensions (‘arms’) to any ion-binding scaffold optimise interactions in one or more pockets. Imperfect stacking and lack of polar interactions will lead to residual flexibility and sub-optimal potency. This seems to be the case for the three diketo compounds, which do not exhibit very well ordered, full occupancy binding modes. On the other hand the binding mode of EGCG is well-defined although possible interactions with some of the hydroxyls in the compound are not fully exploited.

### Resistance

The question of likelihood of resistance is of critical importance in any consideration of anti-viral compounds. This is especially true of influenza viral protein targets which exhibit extensive host and strain dependent sequence variations. For example, in PA-Nter, natural sequence variants are reported in at least 20 of the 200 positions (Figure 1A, see also http://www.ncbi.nlm.nih.gov/genomes/FLU/Database/nph-select.cgi?go=database). On the other hand we note that all the principle residues interacting with the various compounds described here (Tyr24, Glu26, Lys34, Ala37, Ile38, Arg84, Phe105, Tyr130 and Lys134) are highly conserved amongst all influenza A strains, although four of them are substituted in influenza B strains (Phe24, Met34, Asn37, Tyr105), two of them (positions 34 and 37) non-conservatively (Figure 1A). Thus it is likely that there are severe constraints against mutation of these residues whilst retaining carefully tuned enzymatic activity. However, this needs to be explored further given the experience with neuraminidase inhibitors, which also target a conserved enzymatic active site, and to which resistance has naturally emerged.

There is currently very little information available on resistance mutants to polymerase inhibitors partly because they have not been in clinical use. However, it is interesting to note that a mutant was selected by serial passage of influenza strain A/PR/8/34 that is 2–3 times less susceptible to an inhibitor denoted L-742,001 that is very similar to R05-2 but with the cyclohexane group replaced by a benzene group [17]). L-742,001 is reported to have an IC50 of ∼4 µg/mL in a plaque assay. A/PR/8/34 has a threonine at position 20 (Figure 1A) and this became an alanine in the partly resistant mutant. In fact, position 20 is an alanine in the pH1N1 strain used in this work and in most recently circulating A strains. These observations can be explained by the fact that the cyclohexane group of R05-2 that stacks on Tyr24 in pocket 3 is also in close proximity to the side-chain of Ala20 which precedes Tyr24 by one turn in helix α2 (Figure 2C). The methyl-group of a threonine at position 20 would likely enhance the van der Waals contact with the equivalent benzene moiety of L-742,001 by prolonging the hydrophobic platform formed by Tyr24, thus slightly increasing the affinity for the inhibitor. The absence of such additional stabilising interactions probably explains why the electron density for this arm of R05-2 is generally weak. In the accompanying paper the crystal structure of the complex of L-742,001 with loop-deleted A/Vietnam/1203/2004 (H5N1) PA-Nter is reported [24]. Surprisingly, the configuration of L-742,001 is quite different from that we observe for R05-2. The chlorobenzene arm of L-742,001 is rotated 180° to coincide with cyclohexane arm of R05-2 and the benzene arm of L-742,001 enters a different pocket (denoted pocket 5). This orientation of L-742,001 is incompatible with the electron density of R05-2. Despite this difference, which is reminiscent of the promiscuity observed for R05-3, similar conclusions about the role of position 20 in modulating affinity for L-742,001 have been drawn [24], since both compounds have arms entering pocket 3. This example illustrates how detailed structural knowledge about the mode of inhibitor binding, combined with the extensive database of variation in influenza viral proteins, will be extremely useful in designing new inhibitors minimally susceptible to resistance at least from natural mutants known to be viable.

### Concluding remarks

The 2009 H1N1 influenza pandemic [12], the on-going threat to humans of highly pathogenic H5N1 avian influenza viruses [11] and the widespread occurrence of resistance to current anti-influenza drugs [13] has highlighted the need for alternative therapeutic options to treat influenza infections when vaccines are unavailable [14]. Influenza virus can potentially be targeted by antiviral drugs at numerous points in its infectious life cycle [8]. The unique and essential cap-snatching mechanism of transcription by influenza virus polymerase, and in particular, the endonuclease activity, has long been recognised as a good target for antiviral drug development since, firstly, its inhibition could directly stall viral replication at the primary transcription level, secondly, the relevant active sites are likely to be highly conserved across strains and thirdly, the mechanism has no host cell counterpart [15]. Over a nearly twenty year period a number of specific inhibitors of the endonuclease activity have been published (see introduction) although, apparently, none have sufficiently potent anti-viral activity to have entered clinical development. It is interesting to note, however, that the HIV integrase inhibitor Raltegravir, now in clinical use, is also a diketobutanoic acid derivative and targets a two cation containing active site with some similarities to the influenza endonuclease [35]–[37].

The results presented here and in the accompanying paper [24] provide the first high-resolution structural information showing the different binding modes of distinct small molecule metal chelating scaffolds to the active site of the PA endonuclease domain of influenza polymerase. The active site cavity provides multiple distinct pockets capable of accommodating specific extensions to basic metal binding scaffold. However the endonuclease inhibitors analysed here each demonstrated sub-optimal utilisation of the available binding pockets and no one inhibitor sampled all available binding pockets. Furthermore the plasticity of certain regions of the active site cavity, notably the loop containing Tyr24 resulting in induced fit binding by most of the inhibitors, for instance to promote stacking of Tyr24 on aromatic moieties of the compounds. These considerations will be important in guiding modelling and medicinal chemistry approaches to optimization of lead compounds for more efficient inhibition of PA endonuclease. Together with additional endonuclease-inhibitor crystal structures and taking into account known sequence variations that could cause resistance, this will significantly advance the goal of developing novel and efficacious influenza therapeutics that directly target viral replication.

## Methods

### Cloning, expression and purification of pH1N1 PA endonuclease domain

The DNA coding for PA-N-ter (residues 1–198) from A/California/04/2009-pH1N1 was synthesized and sub-cloned in the expression vector pESPRIT002 (EMBL) by GeneArt, (Regensburg, Germany). The sequence was designed to contain an MGSGMA polypeptide linker between the tobacco etch virus (TEV) cleavage site and the N-terminus to obtain 100% cleavage by TEV protease, as used previously [5]. The sequence of A/California/04/2009-H1N1 used is:

mgsgma(1)MEDFVRQCFNPMIVELAEKAMKEYGEDPKIETNKFAAICTHLEVCFMYSDFHFIDERGESIIVESGDPNALLKHRFEIIEGRDRIMAWTVVNSICNTTGVEKPKFLPDLYDYKENRFIEIGVTRREVHIYYLEKANKIKSEKTHIHIFSFTGEEMATKADYTLDEESRARIKTRLFTIRQEMASRSLWDSFRQSERGE -198

To potentially improve crystallisation properties, a deletion of part of the flexible loop (52–73) was engineered by site directed mutagenesis. For this, a PCR amplification of the whole vector containing the wild type gene was performed using two primers flanking the mutation site, one of them phosphorylated, and TurboPfu polymerase (Stratagene). Subsequently, template vector was digested with DpnI (New England Biolabs) and the mutated vector was re-ligated. In the mutant amino acid sequence 52–64 (HFIDERGESIIVE) was replaced by a single glycine.

Wild type and mutant plasmids were transformed to *E. coli* BL21(DE3) (Stratagene) and the protein was expressed in LB medium overnight at 20°C after induction at an OD 0.8–1.0 with 0.2 mM isopropyl-β-thiogalactopyranoside (IPTG). The protein was purified by an immobilized metal affinity column (IMAC). A second IMAC step was performed after cleavage by His-tagged TEV protease, followed by gel filtration on a Superdex 75 column (GE Healthcare). Finally, the protein was concentrated to 10–15 mg.mL^−1^.

### Compounds

Compounds used for co-crystallisation are given in Table 1. DPBA was purchased from Interchim and rUMP and dTMP from Sigma. Compounds R05-01/02/03 (first described in [15]) were custom re-synthesised by Shanghai ChemPartner. EGCG was purchased from Sigma (E4143).

### FRET endonuclease assay

For the fluorescence resonance energy transfer (FRET) assay, influenza A virus A/Victoria/3/1975(H3N2) PA-Nter fragment was purified as described [5] and stored in aliquots at −20°C in buffer containing 20 mM Tris pH 8.0, 100 mM NaCl and 10 mM β-mercaptoethanol. A 20 base dual-labelled RNA oligonucleotide with 5′-FAM (5′carboxyfluorescein) fluorophore and a 3′-BHQ1 quencher (3′-Black Hole Quencher 1) (Sigma) was used as a substrate for the endonuclease. Cleavage of the RNA liberates the fluorophore from the quencher resulting in an increase of the fluorescent signal. All assay components were diluted in assay buffer containing 20 mM Tris-HCl pH 8.0, 100 mM NaCl, 1 mM MnCl2, 10 mM MgCl2 and 10 mM β-mercaptoethanol. The test compounds were dissolved in DMSO and dilution series were prepared in assay buffer resulting in a final plate well DMSO concentration of 0.5%. Each dilution was tested in quadruplicates. Five µl of each compound dilution was provided in the wells of white 384-well microtiter plates (PerkinElmer). After addition of PA-Nter (1 µM final) the plates were sealed and incubated for 30 min at room temperature prior to the addition of 1.6 µM RNA substrate. Then, the increasing fluorescence signal due to RNA cleavage was measured for 50 min in a microplate reader (Synergy HT, Biotek) at 485 nm excitation and 535 nm emission wavelength. The kinetic read interval was 35 sec at a sensitivity of 35. Fluorescence signal data over a period of 20 min were used to calculate the initial velocity (v0) of substrate cleavage for each compound concentration. The IC50 value was determined using a 4-parameter equation (using Graphpad Prism) whereby positive and negative controls were included to define the top and bottom of the curve.

### Antiviral assay – CPE reduction

Influenza A virus was obtained from the American Tissue Culture Collection (A/Aichi/2/68 (H3N2); VR-547). Virus stocks were prepared by propagation of virus on Mardin-Darby canine kidney cells (MDCK; ATCC CCL-34) and infectious titres were determined by 50% tissue culture infective dose (TCID50) analysis. MDCK cells were seeded in 96-well plates at 2×10 E4 cells/well using DMEM/Ham's F-12 (1∶1) medium containing 10% foetal bovine serum (FBS), 2 mM L-Glutamine and 1% antibiotic-antimycotic solution (10.000 Units/ml penicillin, 10 mg/ml streptomycin sulphate, 25 µg/ml amphotericin B) (all from PAA). Until infection the cells were incubated for 5 h at 37°C and 5.0% CO2 to form an 80% confluent monolayer. Test compounds were dissolved in DMSO and dilution series were prepared in infection medium (DMEM/Ham's F-12 (1∶1) containing 5 µg/ml trypsin, and 1% antibiotic-antimycotic solution) resulting in a final plate well DMSO concentration of 1%. The virus stock was generally diluted in infection medium (DMEM/Ham's F-12 (1∶1) containing 5 µg/ml Trypsin, 1% DMSO, and 1% antibiotics) to a theoretical multiplicity of infection (MOI) of 0.05. After removal of the culture medium and a washing step with PBS, virus and compound were added together to the cells. In the wells used for cytotoxicity determination (uninfected cells), the virus suspension was replaced by infection medium. Each treatment was conducted in two replicates. After incubation at 37°C and 5% CO2 for 48 h, each well was observed in the microscope for apparent cytotoxicity, precipitate formation, or other notable abnormalities. Then, cell viability was determined using CellTiter-Glo luminescent cell viability assay (Promega). The supernatant was removed carefully and 65 µl of the reconstituted reagent were added to each well and plates were incubated for 15 min at room temperature under gentle shaking. Then, 60 µl of the solution was transferred to an opaque plate and luminescence (RLU) was measured using Synergy HT plate reader (Biotek). The compounds were titrated on virus infected MDCK cells and the response (RLU) was used to determine the IC50 value using a 4-paramenter equation whereby top and bottom of the curve were defined by the RLU of untreated uninfected cells and untreated infected cells, respectively. The CC50 value was obtained by titrating the compounds on uninfected MDCK cells and similarly fitting the response but with the top of the curve being defined by the RLU of untreated uninfected cells.

### Thermal stabilization assays

The thermal stabilization of the protein in the presence of different inhibitors was performed as described [5], [27]. Briefly, assays were performed with 5 µM H1N1 PA-Nter in 100 mM Hepes pH 7.5, 100 mM NaCl, 1 mM MnCl_2_, 1 mM MgCl_2_, 1 mM DTT in the presence or absence of 500 µM of the indicated inhibitors and a 5× dilution of SYPRO Orange dye (Invitrogen. The dye was excited at 490 nm and the emission light was recorded at 575 nm while the temperature was increased by increments of 1°C per minute from 45–93°C (25 to 73°C for no ligand). The fluorescence versus temperature was graphed in Excel and the inflection point taken as the Tm.

### Crystallization

Initial sitting drop screening was carried out at 20°C mixing 100 nL of protein solution (15 mg.mL^−1^) with 100 nL of reservoir solution using a Cartesian robot. Subsequently, larger crystals were obtained at 20°C by the hanging drop method mixing protein and reservoir solutions in a ratio of 1∶1. The protein solution contained 10–15 mg.mL^−1^ of PA-Nter in 20 mM HEPES pH 7.5, 150 mM NaCl, 2 mM MnCl_2_, 2 mM MgCl_2_. The refined reservoir compositions for native crystals and co-crystallization with different ligands are listed in Table 1. Native crystals and those co-crystallized with DBPA, R05-3 and EGCG were flash frozen in liquid nitrogen after cryo-protection in their reservoir solution containing 25% glycerol. Co-crystals with dTMP or rUMP were frozen in their reservoir solution containing 20% glycerol and 10 mM dTMP or rUMP, respectively. Structures of R05-2 and R05-1 were obtained by soaking co-crystals of PA-N-ter and dTMP or rUMP for 2 h with reservoir solution containing the inhibitor followed by cryo protection in reservoir solution containing 20% glycerol and the inhibitor.

### Crystal structure determination

Diffraction data were collected at 100 K on various beamlines at the European Synchrotron Radiation Facility (Table 3). Datasets were integrated with XDS [38] and scaled with XSCALE. Subsequent data analysis was performed with the CCP4i programme suite. The initial pH1N1 PA N-ter structure was solved by molecular replacement with PHASER [39] using the previously determined H3N2 PA N-ter structure (PDB code 1W69) [5]. Subsequent co-crystal structures were determined with PHASER using the pH1N1 structure. Refinement was carried out with REFMAC [40] and model building with COOT. In the *C*2 and *P*2_1_2_1_2_1_ crystal forms, there are four molecules per asymmetric unit. However because of structural variations between the molecules due to plasticity, in particular the 53–73 region, and the generally good resolution, NCS restraints were not applied. In virtually all structures, residues 139–141 and 196–198 are poorly ordered.

Anomalous scattering from manganese was readily observed for the *P*2_1_2_1_2_1_ and *P*6_2_22 crystal forms at the X-ray energies of normal data collection (∼0.9 Å, Table 3). The signal was much weaker for the *C*2 crystal form (native and DPDA data), probably due to the lower symmetry and hence less redundant data. A separate dataset of the native C2 crystals was collected at a wavelength of 1.55 Å to enhance the anomalous scattering from manganese (K-edge at 1.90 Å) (see main text).

The sequence alignment in Figure 1 was drawn with ESPript (http://espript.ibcp.fr/ESPript/cgi-bin/ESPript.cgi) [41]. Structure figures were drawn with MOLSCRIPT [42] or BOBSCRIPT [43] and rendered with Raster3D [44].

### Data deposition

Structure factors and co-ordinates have been deposited in the PDB as follows: Native (4AVQ), DPBA (4AWF), R05-1 (4AWK), R05-2 (4AVG), R05-3 (4AWG), EGCG (4AWM), dTMP (4AVL), rUMP (4AWH).

## Supporting Information

Figure S1 **Biophysical characterisation of inhibitor binding.** A. **FRET assay for endonuclease inhibition by ECGC**. Example time course of fluorescence signal in the FRET assay for different concentrations of EGCG (see methods). B. **Determination of IC50 for EGCG from FRET data**. Initial velocity values extracted from fluorescence data as in panel A were fitted as described in the methods to extract IC50. Data points show mean and standard error for four parallel experiments. C. **Thermal shift assay for diketo compounds**. Thermal shift assays were performed with 5 µM H1N1 PA-Nter in 100 mM Hepes pH 7.5, 100 mM NaCl, 1 mM MnCl_2_, 1 mM MgCl_2_, 1 mM DTT in the presence or absence of 500 µM of the indicated inhibitors and a 5× dilution of SYPRO Orange dye (Invitrogen) as described [5]. The dye was excited at 490 nm and the emission light was recorded at 575 nm while the temperature was increased by increments of 1°C per minute from 45–93°C (25 to 73°C for no ligand). The estimated Tm values are 53.5, 65, 69, 71 and 69°C for respectively no ligand, DPBA, R05-01, R05-02 and R05-03.
(TIF)

Figure S2 **Electron densities for inhibitors bound in pH1N1 PA endonuclease.** Manganese ions are pink spheres and co-ordinating water molecule blue spheres. Ion co-ordination is shown with green lines. Blue contour: final 2Fo-Fc electron density at 1.0 σ. Brown contour: Fo-Fc unbiased difference map at 2.7 or 2.8 σ (i.e. before inclusion of compound in the model). **A:** DPBA. Yellow contour: anomalous density at 3.0 σ. **B:** R05-03 in the A, B chains in asymmetric unit. Yellow contour: anomalous density at 2.7 σ **C:** R05-03 in the D, C chains in asymmetric unit. Yellow contour: anomalous density at 2.7 σ **D:** R05-02. Yellow contour: anomalous density at 3.0 σ **E:** R05-01. Yellow contour: anomalous density at 5.0 σ. **F:** dTMP. Yellow contour: anomalous density at 4.0 σ.
(TIF)

Figure S3 **EGCG in the active site of PA endonuclease. A:** Electron density for EGCG bound in pH1N1 PA endonuclease. Manganese ions are pink spheres. Blue contour: final 2Fo-Fc electron density at 1.0 σ. Brown contour: Fo-Fc unbiased difference map at 2.7 or 2.8 σ. Yellow contour: anomalous density at 2.7 σ. **B:** Bound EGCG, the divalent cations (two manganese ions, pink spheres) and key active site residues that interact with the compound or are close to it. Putative hydrogen bonds (<3.2 Å) are shown as green dotted lines, and additional possible interactions (<3.6 Å) as blue dotted lines.
(TIF)

Figure S4 **Comparison of pH1N1-rUMP structure with equivalent structure for H5N1 endonuclease (PDB 3HW3).** Protein residues are shown in yellow, rUMP in violet, manganese ions are pink spheres, water molecules as blue spheres and the ion co-ordination is shown with green dotted lines. **A:** Bound rUMP showing stacking of the base on Tyr24 and hydrogen bonding to Lys34. **B:** H5N1 PA with bound rUMP as drawn from PDB entry 3HW3 [24] with the protein in the same orientation as A. In this structure, a water molecule replaces Mn1 and a magnesium ion replaces Mn2. The nucleotide is in a quite different orientation and makes no direct interactions with Tyr24 or Lys34.
(TIF)
