# Supplementary figures and images for: Structural Analysis of Specific Metal Chelating Inhibitor Binding to the Endonuclease Domain of Influenza pH1N1 (2009) Polymerase
Source: PLoS Pathog. 2012 Aug 2;8(8):e1002831. doi: 10.1371/journal.ppat.1002831 (PMC3410856; doi:10.1371/journal.ppat.1002831)

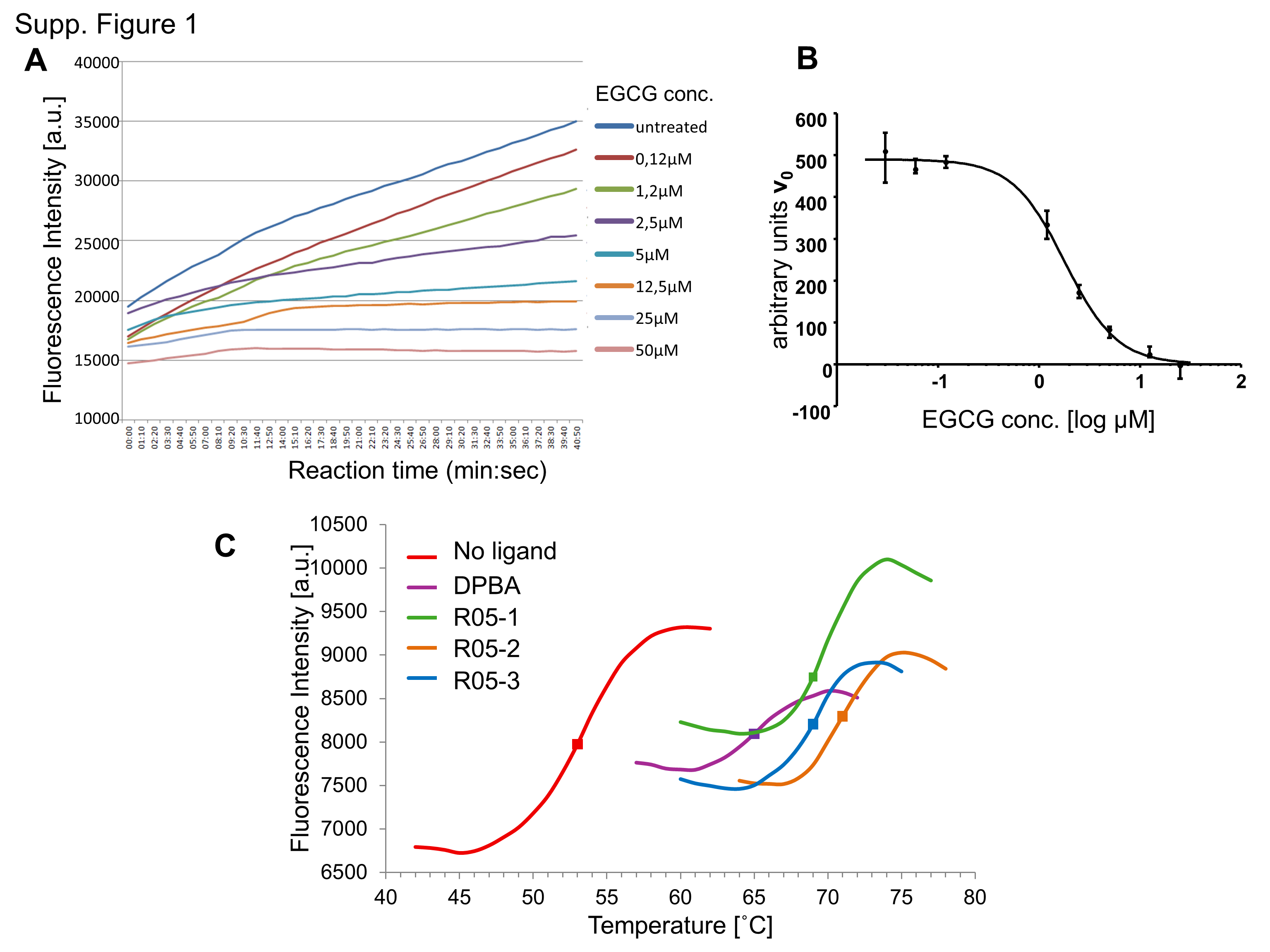

Supplement: Figure S1 — Biophysical characterisation of inhibitor binding. A. FRET assay for endonuclease inhibition by ECGC. Example time course of fluorescence signal in the FRET assay for different concentrations of EGCG (see methods). B. Determination of IC50 for EGCG from FRET data. Initial velocity values extracted from fluorescence data as in panel A were fitted as described in the methods to extract IC50. Data points show mean and standard error for four parallel experiments. C. Thermal shift assay for diketo compounds. Thermal shift assays were performed with 5 µM H1N1 PA-Nter in 100 mM Hepes pH 7.5, 100 mM NaCl, 1 mM MnCl2, 1 mM MgCl2, 1 mM DTT in the presence or absence of 500 µM of the indicated inhibitors and a 5× dilution of SYPRO Orange dye (Invitrogen) as described [5]. The dye was excited at 490 nm and the emission light was recorded at 575 nm while the temperature was increased by increments of 1°C per minute from 45–93°C (25 to 73°C for no ligand). The estimated Tm values are 53.5, 65, 69, 71 and 69°C for respectively no ligand, DPBA, R05-01, R05-02 and R05-03. (TIF) [file ppat.1002831.s001.tif]

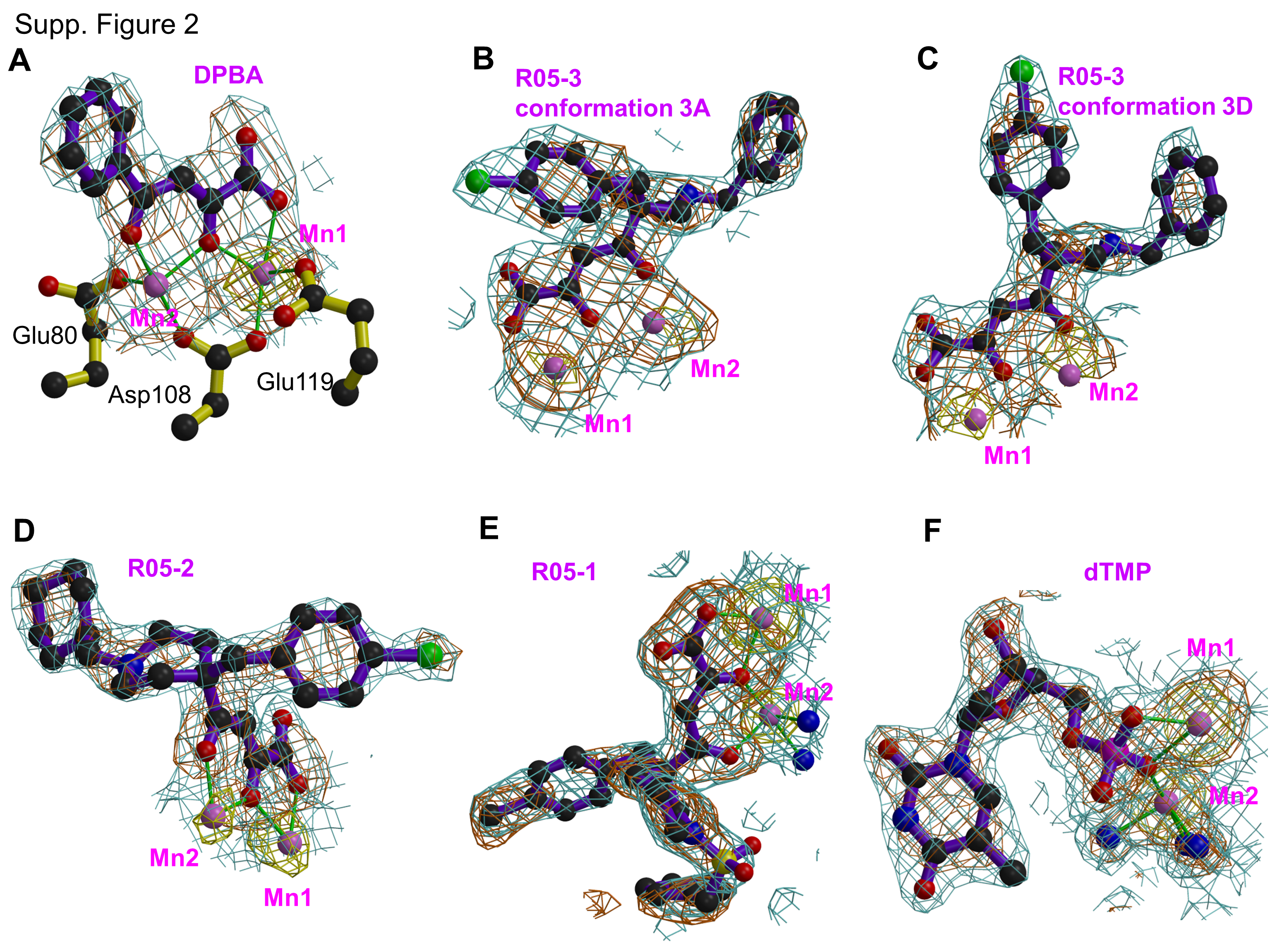

Supplement: Figure S2 — Electron densities for inhibitors bound in pH1N1 PA endonuclease. Manganese ions are pink spheres and co-ordinating water molecule blue spheres. Ion co-ordination is shown with green lines. Blue contour: final 2Fo-Fc electron density at 1.0 σ. Brown contour: Fo-Fc unbiased difference map at 2.7 or 2.8 σ (i.e. before inclusion of compound in the model). A: DPBA. Yellow contour: anomalous density at 3.0 σ. B: R05-03 in the A, B chains in asymmetric unit. Yellow contour: anomalous density at 2.7 σ C: R05-03 in the D, C chains in asymmetric unit. Yellow contour: anomalous density at 2.7 σ D: R05-02. Yellow contour: anomalous density at 3.0 σ E: R05-01. Yellow contour: anomalous density at 5.0 σ. F: dTMP. Yellow contour: anomalous density at 4.0 σ. (TIF) [file ppat.1002831.s002.tif]

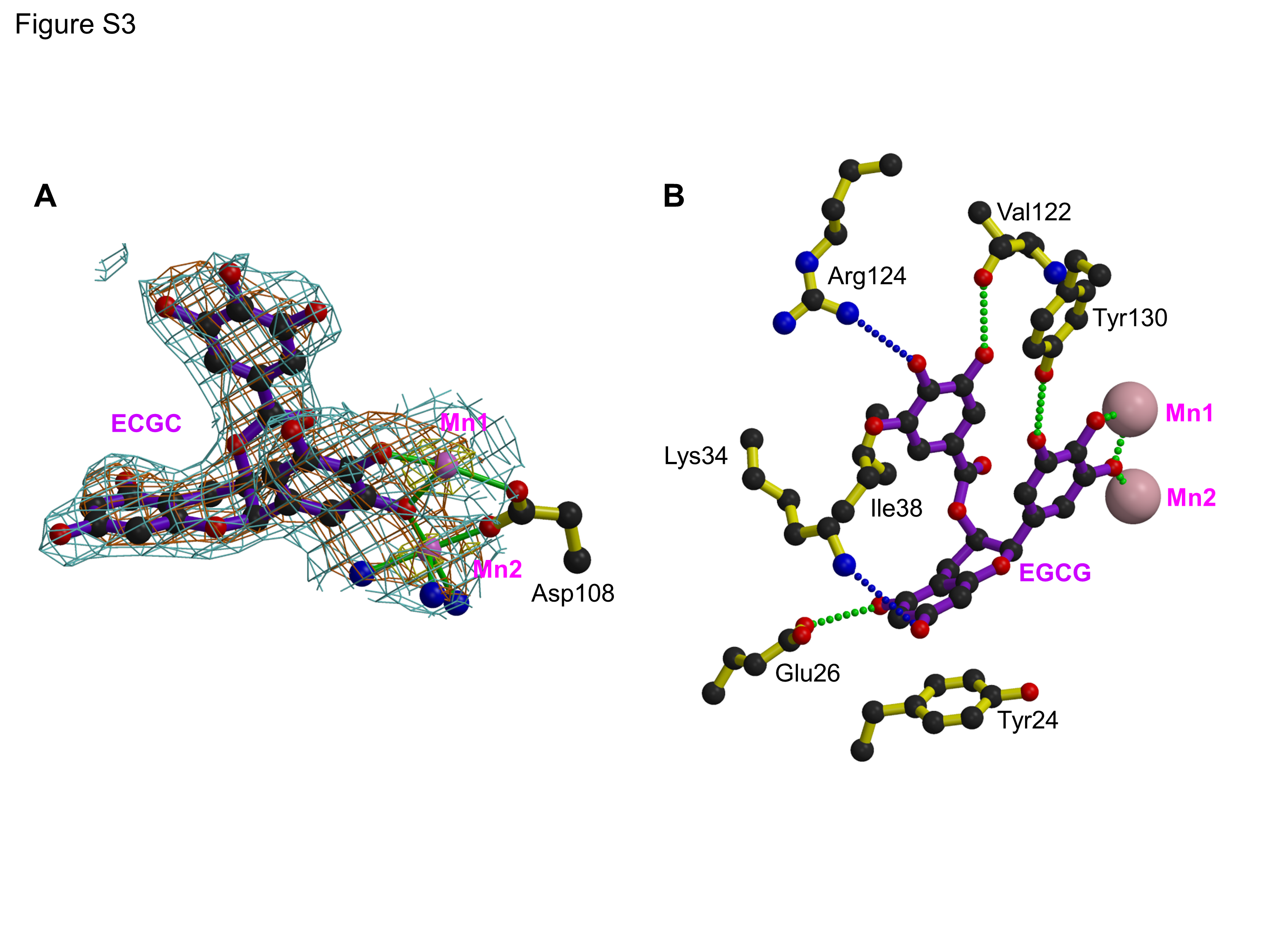

Supplement: Figure S3 — EGCG in the active site of PA endonuclease. A: Electron density for EGCG bound in pH1N1 PA endonuclease. Manganese ions are pink spheres. Blue contour: final 2Fo-Fc electron density at 1.0 σ. Brown contour: Fo-Fc unbiased difference map at 2.7 or 2.8 σ. Yellow contour: anomalous density at 2.7 σ. B: Bound EGCG, the divalent cations (two manganese ions, pink spheres) and key active site residues that interact with the compound or are close to it. Putative hydrogen bonds (<3.2 Å) are shown as green dotted lines, and additional possible interactions (<3.6 Å) as blue dotted lines. (TIF) [file ppat.1002831.s003.tif]

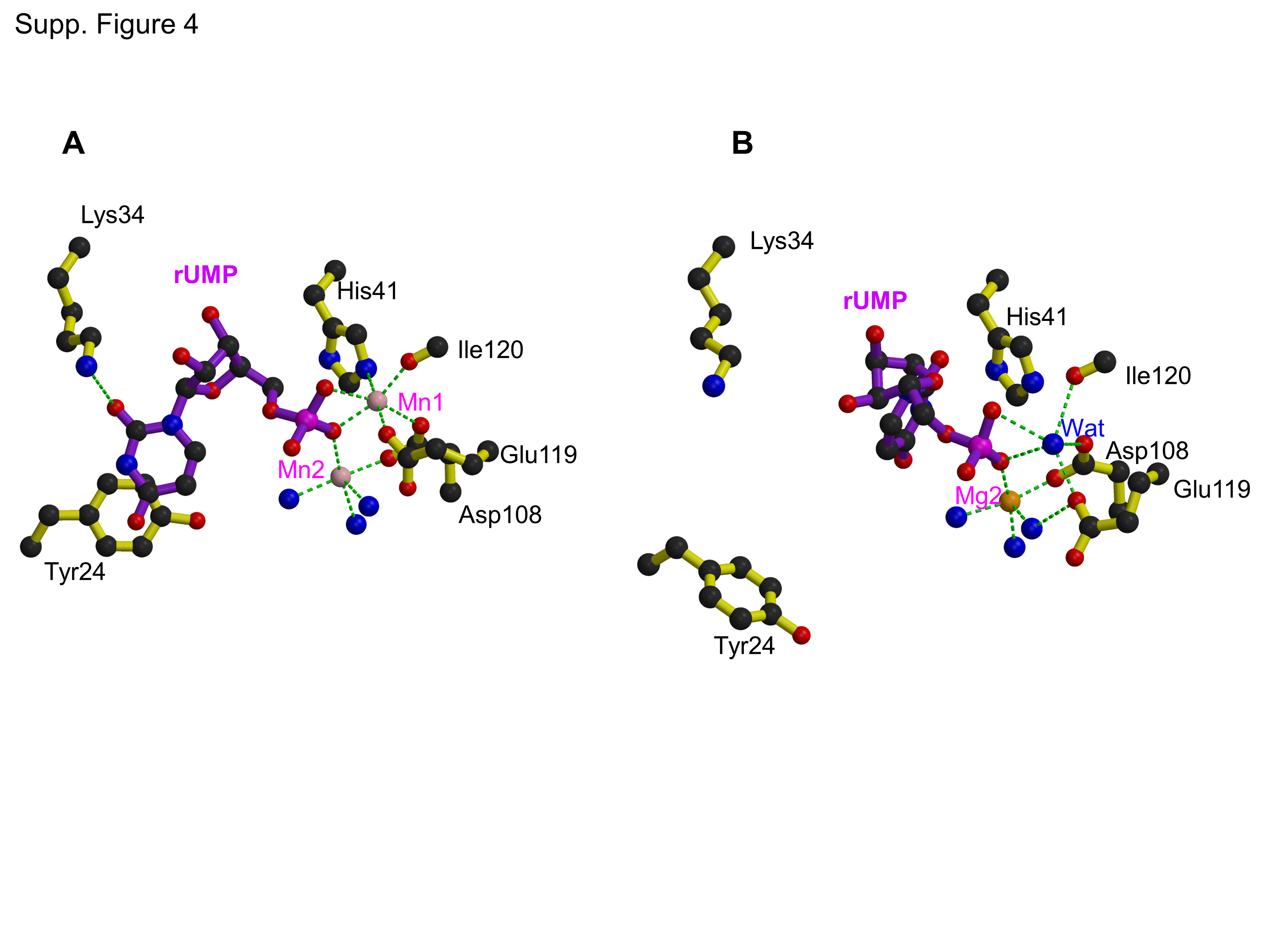

Supplement: Figure S4 — Comparison of pH1N1-rUMP structure with equivalent structure for H5N1 endonuclease (PDB 3HW3). Protein residues are shown in yellow, rUMP in violet, manganese ions are pink spheres, water molecules as blue spheres and the ion co-ordination is shown with green dotted lines. A: Bound rUMP showing stacking of the base on Tyr24 and hydrogen bonding to Lys34. B: H5N1 PA with bound rUMP as drawn from PDB entry 3HW3 [24] with the protein in the same orientation as A. In this structure, a water molecule replaces Mn1 and a magnesium ion replaces Mn2. The nucleotide is in a quite different orientation and makes no direct interactions with Tyr24 or Lys34. (TIF) [file ppat.1002831.s004.tif]
